# Clinical, Functional, and Biological Correlates of Cognitive Dimensions in Major Depressive Disorder – Rationale, Design, and Characteristics of the Cognitive Function and Mood Study (CoFaM-Study)

**DOI:** 10.3389/fpsyt.2016.00150

**Published:** 2016-08-26

**Authors:** Bernhard T. Baune, Tracy Air

**Affiliations:** ^1^Discipline of Psychiatry, School of Medicine, University of Adelaide, Adelaide, SA, Australia

**Keywords:** depression, cognitive dimensions, cognitive function, emotion processing, social cognition, biological correlates, genomic biomarkers

## Abstract

Cross-sectional and longitudinal studies exploring clinical, functional, and biological correlates of major depressive disorder are frequent. In this type of research, depression is most commonly defined as a categorical diagnosis based on studies using diagnostic instruments. Given the phenotypic and biological heterogeneity of depression, we chose to focus the phenotypic assessments on three cognitive dimensions of depression including (a) cognitive performance, (b) emotion processing, and (c) social cognitive functioning. Hence, the overall aim of the study is to investigate the long-term clinical course of these cognitive dimensions in depression and its functional (psychosocial) correlates. We also aim to identify biological “genomic” correlates of these three cognitive dimensions of depression. To address the above overall aim, we created the Cognition and Mood Study (CoFaMS) with the key objective to investigate the clinical, functional, and biological correlates of cognitive dimensions of depression by employing a prospective study design and including a healthy control group. The study commenced in April 2015, including patients with a primary diagnosis of a major depressive episode of major depressive disorder or bipolar disorder according to DSM-IV-TR criteria. The assessments cover the three cognitive dimensions of depression (cognitive performance, emotion processing, and social cognition), cognitive function screening instrument, plus functional scales to assess general, work place, and psychosocial function, depression symptom scales, and clinical course of illness. Blood is collected for comprehensive genomic discovery analyses of biological correlates of cognitive dimensions of depression. The CoFaM-Study represents an innovative approach focusing on cognitive dimensions of depression and its functional and biological “genomic” correlates. The CoFaMS team welcomes collaborations with both national and international researchers.

## Introduction

The lifetime prevalence of 15% for major depressive disorder (MDD) within the general population (1) is among the highest among all mental disorders. MDD is also one of the leading causes of disability and has been estimated to affect 300 million people worldwide (2, 3) and is associated with significant medical and psychosocial morbidity (4–7). Apart from symptoms of impaired mood, major depressive disorder is characterized by additional emotional, psychological, behavioral, physical, and cognitive symptoms, such as neuropsychological dysfunction (8, 9). According to the *Diagnostics and Statistical Manual of Mental Disorders, Fifth Edition* (DSM-5) (10), and the *International Classification of Diseases* (ICD-10) (11), cognitive symptoms of MDD are characterized by reduced concentration, memory deficits, and impaired decision-making processes. Cognitive dysfunction is not necessarily a symptom of aging with depression, but a symptom that may occur frequently across various cognitive domains in patients with first episode of depression and in young MDD patients (15–25 years of age) (12), in depression across all age groups, and during acute and remitted stages of the disorder (9).

Specifically, numerous clinical cross-sectional studies have established that a range of cognitive symptoms occur frequently during the acute state of depression (9). Patients with acute depression show deficits in various domains such as, but not limited to, executive functioning (13–19), audio–verbal functioning (20), memory (21, 22), attention (23), attentional set-shifting (24, 25), visuo-spatial processing, and psychomotor speed (26). A recently published meta-analysis confirmed significant moderate cognitive deficits in the domains of executive function, memory, and attention in patients with depression when compared to controls (27). Additionally, significant correlations between depression severity and cognitive performance were found in the domains of episodic memory, executive function, and processing speed but not for semantic memory or visuo-spatial memory (28).

However, cognitive impairment may not only affect the individual’s ability to function during acute episodes of the illness, as a large body of literature now suggests that cognitive dysfunction may continue into and after remission (6, 29, 30). As shown in a meta-analysis, significant moderate deficits in executive function and attention were found to persist in patients whose depressive symptoms had remitted, indicating that cognitive impairment occurs separately from episodes of depression (27).

In addition to the cognitive deficits during acute and remitted stages of major depression, deficits in psychosocial and overall functioning in MDD are well recognized and of clinical importance. A prospective study found that patients with MDD or bipolar depressive episodes had moderate to severe impairments in work and home functioning as well as mildly to moderately impaired relational functioning (31). While evidence on the direct relationship between cognitive dysfunction and general functioning is emerging, some first evidence suggests that persistent cognitive deficits in depression may play an important role in patients’ ability to fully recover functionally over time (29, 32). If patients fail to return to work, poor cognitive function may be part of this failure as indicated by a study that showed that only cognitively recovered patients were able to return to work (6). Furthermore, a preliminary study suggests that deficits in executive functioning exert a mediating effect on the relationship between depression and impaired activities of daily living (33). A recently published systematic review confirms the relevance of cognitive function in day-to-day functional activities in MDD (34).

Overall, the implications of poor cognitive function are far reaching. Research suggests that MDD patients with neuropsychological deficits tend to be less compliant with antidepressant treatment (35) and show an increased risk for suicide (36), both of which highlight the clinical importance of neuropsychological deficits in depression. In addition to these clinically relevant cognitive deficits in MDD, other cognitive dimensions of depression, such as emotion processing and social cognitive functioning, can be delineated. These dimensions have long been considered at the core of depressive symptoms, as they can affect not only how patients feel about themselves and others but also how they function on a daily basis and at work. Emotional processing distortions in MDD, namely negative attention bias to sad facial expressions (37) and oversensitivity to negative feedback (38), are key clinical features of depression, which may be present in acute and remitted MDD. Similarly, social cognition, which is the ability to identify, perceive, and interpret socially relevant information in relation to oneself and to others, is an important skill that plays a significant role in successful interpersonal and day-to-day functioning. The difficulties with social interaction observed in major depressive disorder may, at least in part, be due to an altered ability to correctly interpret emotional stimuli and mental states (37) that seem to persist even in remission of MDD, if not responding to intervention.

These cognitive dimensions of depression inherit a number of questions that are clinically important and remain unresolved, such as (A) the longitudinal course of cognitive deficits in MDD, (B) the relationship between cognitive deficits and general functioning, and (C) the question how social cognitive deficits relate to cognitive function deficits in domains, such as memory, attention, and executive function. To address these questions in more depth will help provide an understanding as to the clinical course and etiology of depression, identifying treatment targets of depression more clearly, and will contribute to clinical prevention.

Despite significant advances in the elucidating the neurobiology of depression in recent years, the neurobiological correlates of cognition also warrant further investigation. Since it is undisputed that psychological processes, including cognitive function, have a neuronal representation, several structural and functional alterations related to neuropsychological impairments in patients with mood disorders have been documented *via* neuroimaging (9). In addition to gray matter changes such as atrophy in the prefrontal cortex (PFC), the cingular cortex with prominent changes in the left subgenual cingular cortex (39), the temporal cortex, and the basal ganglia (39), white matter damage has also been documented (40). In particular, the effects of cortisol on brain structure has been suggested to be relevant in the etiology of MDD more generally and of cognitive impairment in depression in particular. Since pathological abnormalities of the hypothalamic-pituitary-adrenal axis (HPA) in conjunction with an increased excretion of cortisol in depression have been described, the abnormalities of the HPA axis following stress may lead, or at least contribute, to the structural changes such as those evident in the hippocampus. These findings are supported by numerous studies, which have established an association between duration of the mood disorder/numbers of episodes (41, 42) and early traumatization (43), and the degree of hippocampal damage. In addition, the effect of stress on the hippocampus appears to be modulated by genetic factors (44). It should also be considered that structural abnormalities can represent a vulnerability factor for the development of mood disorders and hypercortisolism (45). Overall, imaging studies show that neurocircuitry dysfunction appears to occur in the same pathways in depression and cognition (9), similarly to the involvement of monoamines, glutamate, and gamma-aminobutyric acid (GABA) in both depression and cognition.

Additional neurobiological factors that affect both depression and cognition include neuroinflammatory and metabolic changes (46). Specifically, the role of inflammation is an important biological mechanism of cognitive function. This suggestion rests on both the cytokine model of depression (47, 48) and on the the cytokine model of cognitive function that outline the role of cytokines (e.g., TNF-α, IL-6, and IL-1β) in regulating sickness behavior and neurobiological processes subserving cognition, i.e., synaptic plasticity, synaptic scaling, neurogenesis, neurotransmission, and long-term potentiation/depression (LTP/LTD) that are relevant to cognitive function in depression (49). In a recent pilot investigation of CoFaMS, we reported the involvement of B lymphocyte proliferation and ribosomal S26 transcripts specifically in relation to cognitive dysfunction in remitted major depressive disorder (50). Other potential genomic biomarkers of cognitive dysfunction in depression, such as inflammatory and immune, among neurotransmitter and neurotrophic markers require further investigation (51).

Hence, the overall aim of the CoFaM Study is to investigate the long-term clinical course of cognitive dimensions in depression and its functional (psychosocial) correlates. In addition, the study aims to identify biological “genomic” correlates of these cognitive dimensions of depression. By taking a prospective approach and by including a healthy control group, the study will increase the understanding of the cognitive dimensions of depression, its biological correlates, and its functional outcomes, etiology, pathophysiology, and state vs. trait characterization.

## Methods

### Objectives

To address the above overall aim, we created the Cognition and Mood Study (CoFaMS) with the key objective to investigate the clinical, functional, and biological correlates of cognitive dimensions of depression by employing a prospective study design and including a healthy control group.

The key hypotheses and aims of the project are:

*Hypothesis 1*: Participants with mood disorders have poorer neuropsychological function in the cognitive dimensions of cognitive function, emotion processing, and social cognition compared to healthy matched controls.
*Hypothesis 2*: Participants with mood disorders have poorer quality of life and poorer psychosocial functional outcomes that will correlate with the level of impairment in cognitive dimensions of depression.
*Hypothesis 3*: Participants with mood disorders show a biological genomic “signature” that relates to cognitive dimensions of depression.

### Study Design and Recruitment

The outlined study design commenced in April 2015 and includes baseline and follow-up assessments at 6-weeks and 12-months post baseline. The design of the study is prospective in nature and includes a healthy control group. The naturalistic recruitment of patients aged 18 years or above occurs in inpatient and outpatient units through research clinics of the Department of Psychiatry, University of Adelaide and the Eastern, Western, and Northern Mental Health Networks in Adelaide, South Australia. Recruitment may also include the general community through advertisement. Healthy controls are recruited from the same population background as the patients.

### Inclusion and Exclusion Criteria

The inclusion criterion is a current or previous diagnosis of a major depressive episode according to the DSM-IV-TR (52). Primary diagnosis of participants therefore included both unipolar and bipolar depressive disorders. Exclusion criteria were previous diagnosis or high screening score of a psychotic disorder, dementia, learning disorder, eating disorder or autistic spectrum disorder or medical conditions affecting the central nervous system (CNS) (e.g., multiple sclerosis, Parkinson’s disease, brain tumor). Healthy controls were required to have no previous or current psychiatric morbidity.

### Ethics

The study was approved by the Human Research Ethics Committees at the University of Adelaide (approval number: H-160-2011) and the Royal Adelaide Hospital Human Research Ethics (approval number: 111230). Study participants were explained all study details in writing and in person before giving informed consent.

## Clinical, Self-Report, and Cognitive Assessments

### Diagnostic Interview

All participants are screened for mood symptoms using the MINI600 Neuropsychiatric Diagnostic Interview. All sections of the MINI600 are administered, including Major Depressive Episodes, Manic and Hypomanic Episodes, Psychotic Disorders and Mood Disorders with Psychotic features, Panic Disorder, Alcohol Abuse and Dependence, and Substance Abuse and Dependence. The MINI600 is a well-validated measure with psychometrics showing good to excellent specificity and sensitivity concordance to both the Structured Clinical Interview for the American Psychiatric Association Diagnostic criteria (SCID) and the Composite International Diagnostic Interview (ICD-10) (53, 54). The psychometric properties and the psychometric validation of the MINI have been published extensively (53, 54).

### Symptom Severity

The Structured Interview Guide of the Hamilton Anxiety and Depression Scale (SIGH-AD) (55) is administered as it assists with establishing severity of symptoms. The SIGH–AD is a 31-item structured interview that combines the Hamilton Depression Scale (HAM-D, 17 items) and the Hamilton Anxiety Scale (HAM-A, 14 items). Values over 15 represent clinically significant levels of anxiety or depression, respectively.

### Psychiatric History

A psychiatric history checklist is administered to assess information, such as age of onset of illness, number of lifetime episodes of mood disorders, number of hospitalizations, and family history of mental illness.

### Functioning

The participants are administered a questionnaire assessing domains of activities in daily living, using the Functioning Assessment Short Test (FAST) (56, 57). The FAST scale consists of 24 items and has been developed for the clinical evaluation of the main difficulties in daily functioning presented by psychiatric patients, particularly, bipolar disorder patients. It is easy to apply, requires only a short period of time for administration, and is available in several languages. The items are rated 0 (no impairment), 1 (mild impairment), 2 (moderate impairment), or 3 (severe impairment). The overall FAST scores range from 0 to 72, where higher scores indicate greater disability, with scores above 11 indicating the presence of significant disability. The FAST derives individual scores for various domains of function, including a global score of functioning and the domains of autonomy, occupation, cognition, finances, relationship, and leisure. The time frame for evaluation of functioning is the last 14 days.

### Work Productivity

A self-made employment questionnaire is administered to the participants. This questionnaire was developed to assess the impact of cognitive problems on employment status and work productivity in individuals suffering from mood disorders. The questionnaire is an interviewer-administered instrument. The studied time frame refers to the current employment status of the participant in the first section and their work productivity over the last 7 days in the second section. It is quick and easy to administer (on average it takes ~5 min) and provides a comprehensive assessment of the impact mood-related cognitive dysfunction has on occupational functioning.

### General Functioning

Participants are rated on their symptom severity using the Clinical Global Impression Severity Scale (CGI-S) and the Global Assessment of Functioning (GAF). The Clinical Global Impression scale (CGI) is frequently used in clinical research because of its face validity and practicability. The GAF combines the evaluation of symptoms as well as relational, social, and occupational functioning on a single axis. The scale runs from 1 to 100 and is divided into 10 equal parts providing defining characteristics, both symptoms and functioning, for each 10-point interval. A low rating reflects worse symptoms and a poorer level of functioning, whereas a high rating reflects less symptoms and a better level of functioning.

### Treatment Response to Psychotropic Medication

The Lifetime Psychotropic Treatment Response scale (LPTR) is a modified scale from the Lithium Lifetime Treatment Response scale (LLTR) (58). The LPTR scale covers treatment response to various types of psychotropic medication and hence replaces the LLTR. Criterion A is used to estimate response to antidepressant treatment, while Criterion B is used to establish whether there is a causal relationship between clinical improvement and the treatment. The scale is quick and easy to administer (on average it takes about 5 min) and gives an overall picture of the effectiveness of antidepressant medication.

### Assessments of Additional Clinical Characteristics

Participants will complete a battery of self-report questionnaires using standardized scales designed to assess aspects of emotion processing, stress and maltreatment exposure, perceptions of stress, coping strategies, quality of life, general capacity to function, health service utilization, and health beliefs. All components of the self-report measure are derived from well validated and widely used measures that are available in the public domain. Self-report measures include the Positive and Negative Affect Schedule (PANAS) (59–64), Perceived Stress Scale (PSS) (65–68), Short Form Health Survey (SF-36) (69–75), the Childhood Trauma Questionnaire (CTQ) (76), the Center for Epidemiologic Depression Scale (CES-D) (77), the Functional Assessment of Cancer Therapy Cognition (FACT-Cog version 3) (78), and the Resilience Scale (79). The psychometric properties of these self-report measures have been published widely.

Some participants that will be recruited to the study will have a history of, or are suffering from depression with peripartum onset. These participants, due to their role as mothers of babies and very young children, face specific requirements of daily functioning, which are not well captured in the functioning scales used otherwise in the study. To address this potential gap, female participants with young children aged 0–5 are given the following questionnaires.

#### The Parenting Scale

The Parenting Scale (80) is a 30-item self-report questionnaire that measures three dysfunctional discipline styles in parents: laxness (permissive discipline), over-reactivity (authoritarian discipline), and hostility (use of verbal or physical force). Each item requires the parent to rate the likelihood of using a particular discipline strategy in response to common child misbehaviors using a 7-point Likert-type scale. Item scares are summed and then averaged to give a total score ranging from 1 to 7. The Parenting Scale has been found to be a valid and reliable tool with good test–retest reliability (*r* = 0.84) and is recommended as a tool for measuring parenting skill (81) and will be an important and relevant measure of functioning specifically in mothers of young children. On average this questionnaire takes 10 min.

#### The Parenting Tasks Checklist

The Parenting Tasks Checklist (82) consists of 28 items (self-report) designed to assess task-specific self-efficacy in parents. Parents rate how confident they feel when they are dealing with difficult child behavior in common parenting situations. Confidence is rated on a scale from 0 (“certain I cannot do it”) to 10 (“certain I can do it”). Two dimensions are measured: behavioral self-efficacy (confidence in dealing with specific child behaviors) and setting self-efficacy (confidence in different settings). Both scales (each has 14 items) have been shown to have good internal consistency (82). The emphasis of this test on self-efficacy complements, the functional domains of parenting assessed by the Parenting Scale (see above), and further adds to the detailed functional characterization, specific to life situation, of participating mothers with young children.

#### The Edinburgh Postnatal Depression Scale

The Edinburgh Postnatal Depression Scale (EPDS) is a self-report 10-item questionnaire that was developed to identify women who have post-partum depression (PPD). Generally, the EPDS (83) is used as the gold standard to determine the presence of PPD, and it has been used antenatally to identify women at risk for PPD. The measure takes ~5 min to complete.

### Cognitive Assessments

Participants are administered series of computer-based game-like activities using the Psychology Experiment Building Language (PEBL), designed to assess memory and learning, attention, and working memory, executive function, and social cognition. All tests have been psychometrically validated and are used extensively in cognitive function research. The paper–pencil tests include the Repeatable Battery for the Assessment of Neuropsychological Status (RBANS) (84), the computer-based tests are part of the PEBL Battery *– The PEBL* (85), and the social cognition assessments are elements of the Wechsler Adult Intelligence Scale Advanced Clinical Solutions Package (WAIS-IV-ACS) (86, 87).

#### The Psychology Experiment Building Language

The PEBL is a free, open-source software system that allows researchers and clinicians to design, run, and share among 70 behavioral tests (85). We chose this battery as it is computerized, is available on a range of platforms, is updated frequently, and offers a range of cognitive tests suitable for the purpose of our study. For the CoFaM study, we selected the following cognitive tests from the *PEBL* (88).

##### Tower of London Test

*Tower of London Test* (89) is a well-known test used in applied clinical neuropsychology for the assessment of executive functioning specifically to detect deficits in planning, which may occur due to a variety of medical and neuropsychiatric conditions.

##### Wisconsin Card Sort Test

*Wisconsin Card Sort Test (WCST)* (90) is a neuropsychological test of “set-shifting,” i.e., the ability to display flexibility in the face of changing schedules of reinforcement. Successful completion of the test relies upon a number of intact cognitive functions including attention, working memory, and visual processing.

##### Corsi Block Tapping Test

*Corsi Block Tapping Test* (91) assesses visuo-spatial short term working memory. It involves clicking on and copying a sequence of up to nine identical spatially separated blocks as they are displayed in a certain order. The sequence starts out simple, usually using two blocks, but becomes more complex until the subject’s performance suffers.

##### Stroop Test – Stroop – Color-Word Interference

The Stroop test is a neuropsychological measure assessing processing speed and attention administered by computer (92). This is the only test we are using to assess processing speed. The Stroop interference task, in particular, measures the ease with which a person can shift his or her perceptual set to conform to changing demands. The interference task consists of a series of color words, but the words themselves are colored in a different color ink than the color to which they refer. Participants are required to name the color of the word but not the word itself. Impaired attention/concentration is a common symptom in mood disorders. The administration of this test is about 7–10 min.

##### Repeatable Battery for the Assessment of Neuropsychological Status

The RBANS is a brief, individually administered screening instrument that aids in determining the neuropsychological status of adults aged 20–89 years (84). It has been used in a variety of clinical populations, including depression, dementia, and schizophrenia. The RBANS was developed for the dual process of identifying and characterizing abnormal cognitive decline in older adults and as a screening battery for younger patients. The RBANS gives scaled index scores for five cognitive domains: immediate memory, visuo-spatial/constructional, language, attention, and delayed memory. The RBANS has been demonstrated to have high reliability, test–retest stability, inter-rater reliability, content validity, and construct validity and has been administered in various clinical populations, including schizophrenia, depression, and dementia among others (86, 87).

#### THINC-It Tool

The THINC-it tool has been developed in response to the need for a brief screening instrument for detecting cognitive deficits in patients with depression. It was designed to screen – not to diagnose – for objectively assessed cognitive function in depression by employing a variety of well-established cognitive tests (93). The tool contains tests of digit coding, choice reaction time, the one-back memory paradigm, and various versions of the Trail Making Test Part B. In addition, measure of subjectively reported cognitive deficits – *Perceived Deficit Questionnaire (PDQ)* – has been added to the THINC-it assessment tool (94, 95).

##### Digit Coding

This is a measure of attention, perceptual speed, motor speed, visual scanning, and memory. The test requires the examinee to transcribe a unique geometric symbol with its corresponding number. The examinee is initially shown a key containing the numbers from 1 to 6. Under each number, there is a corresponding geometric symbol. The participant is then shown a series of numbers on the screen and asked to match the number with the corresponding geometric symbol.

##### Choice Reaction Time

This test is used to assess psychomotor speed and choice reaction time. In this test, participants are instructed to respond by pressing keys on the left or the right hand side of the keyboard corresponding to the direction to which an arrow on the screen is pointing.

##### N-Back

The N-Back test is measures executive control of the updating of information in working memory. The N-Back task is one in which the participant is presented a series of stimuli at a constant rate. The task of the participant is to map the currently presented stimulus on arrow keys of a keyboard to one they have recently seen in the stream (one position back).

##### Trail-Making Test B

Trail-Making Test B is a neuropsychological test of visual attention and task switching. Participants are instructed to connect a set of 18 dots as fast as possible while still maintaining accuracy. It can provide information about visual search speed, scanning, speed of processing, mental flexibility, as well as executive functioning.

#### Social Cognition

The WAIS ACS Social Cognition Test is an integrated test including facial affect, prosody, body language, and mental state interpretation. It has the advantage of a large normative sample of 800 subjects matched to the US census and has been validated with promising results against currently used facial affect recognition tasks in the setting of other psychiatric conditions. It comprised three subtests: affect naming, prosody-face matching, and prosody-pair matching. The subgroups each test a different aspect of social cognition (86, 87).

### Blood Specimen

For later genomic biomarker analyses (e.g., genetic, gene expression, sequencing, proteomics, epigenomics, serum/plasma levels of biomarkers), peripheral blood will be taken at each time-point of assessment for deriving DNA, RNA, serum, and plasma. The storage of biomaterials is split between different refrigerators, all of which were monitored by a central alert system 24 h/day 7 days/week.

### Quality Assurance and Data Management

The primary goal of all quality assurance measures is to generate high quality data. The CoFaMS standard operations manual contains operating procedures for all CoFaMS interview and examination components. Prior to the beginning of CoFaMS-Baseline, the members of the study team underwent an initial training with follow-up quality checks. Performance is closely monitored and routinely assessed. Data management has been carried out in parallel with data collection, based on standardized and partly automated procedures for data processing and plausibility checking. Data backup routines are scheduled on a daily basis.

### Biometric Concept and Statistical Analyses

The primary endpoints of COFaMS are the presence and severity of deficits of the cognitive dimensions of depression namely cognitive function, emotion processing, and social cognition and also include psychosocial and general function, genomic markers, and serum/plasma levels of biomarkers. Secondary outcomes include change in cognitive dimensions and psychosocial function over time and genomic markers and serum/plasma levels of biomarkers in peripheral blood.

Depending on the outcome scale level (continuous vs. categorical) and time point of assessment (baseline vs. follow-up), the statistical methods comprise multivariable linear or logistic regression analyses, accounting for time-varying predictors and repeated outcome assessment. In addition, genomic marker analyses require specific software suitable for genetic analyses, gene expression analyses, network analyses, proteomics analyses, and other specialized analysis software packages.

## Discussion

Depression is a mental disorder that is characterized by a recurrent course of illness in many cases, by clinical and biological heterogeneity contributes largest to the burden of disease. Specifically, depression reduces psychosocial function and work productivity. Among the symptom clusters of depression, cognitive dimensions are prominent and include emotion processing, cognitive function, and social cognition. These cognitive dimensions as key features of depression appear to be relevant for psychosocial function and present as targets of psychological treatment (e.g., cognitive-behavioral therapy). The CoFaM Study aims to improve the understanding of the clinical, functional, and biological correlates of three cognitive dimensions of depression: emotion processing, cognitive function, and social cognition. Advances in the understanding of these cognitive dimensions are important to identify new treatment targets and to prevent decline in functional capacity. Recruitment of cases and controls of CoFaMS is ongoing. Study recruitment continues until December 2018.

First, the CoFaM Study is suited to characterize three major cognitive dimensions of depression with an emphasis on cognitive function, social cognition, and emotion processing in depressed participants compared to healthy controls. The important trait-state characteristics of cognitive symptoms of depression will be addressed since this study includes patients with current, recurrent, and remitted depression cross-sectionally and prospectively. In addition, the included assessment of psychiatric comorbidity, such as anxiety disorders, will enable us to account for the impact of psychiatric comorbidity on cognitive dimensions of depression. A comparison to other mood disorders, such as bipolar disorder, extends this approach.

Second, the often observed psychosocial and functional decline and inability to recover after an acute episode of depression to premorbid functioning requires a better understanding of the mediators of functional decline. The CoFaM Study will address this key question by investigating the mediating role of the three cognitive dimensions in functional impairment decline in depression. A possible outcome of this study might be a better understanding of this crucial relationship, and the research might provide new leads into improving psychosocial functioning in the future.

Third, the biological correlates and genomic biomarker of depression specifically are lacking in the field. One of the reasons might be the heterogeneity of depression that inhibits a clearer correlation between symptoms of depression and biological correlates. By focusing on cognitive dimensions of depression, it is assumed that cognitive function might more closely correlate with biological markers (e.g., BDNF, inflammatory marker) than a global syndrome of depression consisting of a variety of symptoms clusters. The CoFaM Study allows for the study of the molecular underpinnings of cognitive dimensions by collecting extensively specimen for genetic, gene expression, epigenomic, sequencing, and serum/plasma analyses.

In conclusion, the CoFaM Study represents an innovative approach focusing on cognitive dimensions of depression and its functional and biological “genomic” correlates. The CoFaMS team welcomes collaborations with both national and international researchers.

## Author Contributions

BB conceived the study, supervised its design, the coordination, and execution of the study. BB drafted the manuscript. TA provided specific knowledge to the execution of the study and made substantial contributions to the acquisition of the data. All authors read and approved the final manuscript.

## Conflict of Interest Statement

The authors declare that the research was conducted in the absence of any commercial or financial relationships that could be construed as a potential conflict of interest.
